# Effects of Internal and External Cues on Brain Activity and Gait in Parkinson’s Disease: Findings From BARC-PD

**DOI:** 10.1177/15459683251351876

**Published:** 2025-07-13

**Authors:** Rodrigo Vitorio, Rosie Morris, Lisa Graham, Julia Das, Richard Walker, Claire McDonald, Martina Mancini, Samuel Stuart

**Affiliations:** 1 Department of Sport, Exercise and Rehabilitation, Northumbria University, Newcastle upon Tyne, UK; 2 Northumbria Healthcare NHS Foundation Trust, North Tyneside General Hospital, Newcastle upon Tyne, UK; 3 Gateshead Health NHS Foundation Trust, Gateshead, UK; 4 Department of Neurology, Oregon Health and Science University, Portland, Oregon, USA

**Keywords:** EEG, fNIRS, gait, rehabilitation

## Abstract

**Background:**

Internal and external cueing strategies are often applied to alleviate gait deficits in Parkinson’s disease (PD). However, it remains unclear which type of cueing strategy is most effective at different disease stages. The underlying neural mechanisms of response to cueing are also unknown.

**Objective:**

To investigate the immediate response of multiple brain cortical regions and gait to internal and external cueing in people at different stages of PD.

**Methods:**

People with PD (n = 80) were split into groups dependent on their disease stage (Hoehn and Yahr [H&Y] stage I to III). Participants performed a baseline walk without cues followed by randomized cued walking conditions (internal and external [visual, auditory and tactile] cues). A combined functional near-infrared spectroscopy (fNIRS) and electroencephalography (EEG) system assessed cortical brain activity while walking. Wearable inertial sensors assessed gait.

**Results:**

Cue-related gait improvements were not influenced by H&Y stage; moderate or large effect sizes were only observed for internal cueing and external visual cueing. fNIRS findings suggested cortical response was similar across H&Y stages, with increased activity in the prefrontal cortex with internal cues; and increased activity in the primary motor and visual cortices with external cues. However, EEG showed that people with PD in H&YIII had higher parietal alpha power than those in H&YI in the auditory, tactile, and visual cueing conditions.

**Conclusion:**

Gait improvement with cueing was similar across PD stages and underpinned by cognitive, motor, and/or sensory neural processing within selective brain regions that may be influenced by PD stage (i.e., parietal cortex).

## Introduction

Gait impairments are debilitating and an early feature of Parkinson’s disease (PD).^1,2^ Gait impairments caused by PD are progressive, affect functional independence and are among the major causes of falls in PD.^2^ Gait impairments in PD are characterized by a shift from automatic to more conscious control of gait,^3,4^ with increased executive-attentional resources required to overcome deficits in subcortical brain regions; and this is thought to be exacerbated in people with freezing of gait (FOG).^5^

Gait impairments in PD lack definitive treatment. Standard antiparkinsonian treatment, dopaminergic medications, has limited effect on gait impairments.^6,7^ Despite improvement in selective parameters (e.g., speed and step length), gait impairments do not resolve completely with medications.^8^ Therefore, there is an unmet need for additional interventions for gait deficts in PD. Implementation of internal (i.e., thinking about taking larger steps) or external (e.g., auditory, tactile or visual sensory stimuli) cueing is a common clinical practice that can improve gait and reduce falls risk.^9-13^

Physical therapy guidelines suggest that different cueing strategies may be useful for gait improvement at specific stages of PD.^14^ However, recommendations are based on subjective clinical judgement and do not thoroughly differentiate cue effectiveness across PD stages. Further, cue recommendations in PD are supported by limited objective evidence. Studies involving mid-late stage (Hoehn and Yahr [H&Y] II-IV) PD participants suggest that in the later stages of PD, when FOG and cognitive deficits may be prevalent, only external cues are effective.^15,16^ Whereas internal cues are thought to be most useful in early-stage disease (H&Y I) when cognition is largely intact. Alternatively, recent evidence has suggested that internal cues may be more effective at improving gait than external cues in mid-late stage PD (H&Y II-III),^17^ which directly contrasts previous physical therapy recommendations. Furthermore, evidence for cueing in early stage PD is lacking. A recent longitudinal pilot study demonstrated that response to auditory cues may be influenced by PD progression, with limited gait benefits or possible cue induced impairments (e.g., increased gait variability) in early PD (i.e., within 6 months of diagnosis: H&Y I-II) but increased gait benefits at later stages (3 years post diagnosis: H&Y II-III).^18^

The neural mechanisms underlying gait response to cueing in PD are poorly understood. This is a contributing factor to the lack of knowledge related to cue implementation stage, as well as reports of variable response^19^ and selective short-term gait characteristic improvement.^20^ For example, lack of response to cues may occur due to the inability to recruit appropriate brain regions to control cued walking. Theories suggest that different cueing strategies may be underpinned by different attentional and/or sensorimotor mechanisms. External auditory cues are suggested to improve gait through replacement of rhythmic basal ganglia output via external stimuli that reduces prefrontal cortex (PFC) burden during gait^21^; and visual or tactile cues may enhance sensory feedback and activate compensatory attentional mechanisms^10^ to allow faster subconscious processing of sensory information when walking.^22,23^ Whereas, internal cueing may use attentional mechanisms that by-pass impaired basal ganglia circuitry to improve gait.^24^ To date however, these theories remain relatively unexplored, likely due to an inability to image the brain during walking. Therefore, development of a robust body of evidence about the brain response to cueing can contribute to the optimization of cueing strategies to alleviate gait impairments in people with PD.

Progression in mobile brain imaging techniques, such as functional near-infrared spectroscopy (fNIRS) and electroencephalography (EEG), have allowed monitoring of brain activity during actual walking. However, studies applying these techniques to understand brain response to cueing in PD are limited. Our recent fNIRS study involving people with PD (with and without FOG) showed no change in prefrontal activity with tactile cues compared to usual walking, which was despite gait improvements with cueing.^25^ However, this study only assessed the PFC. Further, our recent EEG study showed that people with PD reduced beta band power in the central parietal cortex in response to walking with visual cues analysis.^26^ These two previous studies were limited by not comparing different cueing modalities or the influence of disease stage on response to cueing.

The current study is focused on addressing the following gaps: (i) it remains unclear which type of cueing strategy is most effective at different disease stages; (ii) the underlying neural mechanisms of response to cueing are unknown. Therefore, we used a combined EEG/fNIRS system and wearable inertial sensors to investigate the response of multiple brain cortical regions and gait to internal and external cueing in people with PD across different disease stages (i.e., H&Y I to H&YIII). We hypothesized that H&YI would respond more to internal than external cue stimulus, but with the least activation of the prefrontal cortex at this PD stage compared to H&YII and H&YIII. Additionally, we hypothesized that external cue stimulus would elicit multi-region cortical response at H&YIII, with activation of prefrontal, motor and parietal regions dependent upon cue stimulus type.

## Methods

### Study Setting

This trial was carried out at the Clinical Gait Laboratory, Northumbria University, Newcastle upon Tyne.

### Ethical Approval and Registration

This study conforms to the Declaration of Helsinki and has been approved by the London-Bloomsbury NHS Research Ethics Committee (and Health Research Authority; 20/LO/1036, 05/10/2020). Participation in the study was voluntary and required written informed consent from each participant. This trial was prospectively registered at clinicaltrials.gov (NCT04863560; 28 April 2021; https://clinicaltrials.gov/ct2/show/NCT04863560). The study protocol is published elsewhere.^27^

### Recruitment and Eligibility Criteria

Participants were recruited from Movement Disorder Clinics at Northumbria Healthcare NHS Foundation Trust and Gateshead Health NHS Foundation Trust, and through the Parkinson’s UK research excellence network and DeNDRoN Research Case Register.

Inclusion criteria: Clinical diagnosis of PD by a movement disorder specialist according to UK brain bank criteria; H&Y stage I-III; aged >50 years; able to walk and stand unaided; adequate hearing (as evaluated by the whisper test; stand 2 meters behind subject and whisper a 2 syllable word, subject repeats word) and vision capabilities (as measured using a Snellen chart—6/18-6/12); stable medication for the past 1 month and anticipated over a period of 6 months.

Exclusion criteria: Psychiatric co-morbidity (e.g., Schizophrenia, major depressive disorder as determined by geriatric depression scale - GDS-15; <10^28^); clinical diagnosis of dementia or other severe cognitive impairment (Montreal Cognitive Assessment <21^29^); history of neurological disorders other than PD; acute lower back or lower extremity pain, peripheral neuropathy, rheumatic and orthopedic diseases; unstable medical condition including cardiovascular issues in the past 6 months; unable to comply with testing protocol; and interfering research project or therapy.

### Assignment and Blinding

Participants (n = 80) were split into groups dependent on the stage of their disease (classified with H&Y scale, the most widely used scale for classification of PD stages); n = 20 H&YI (early disease, mild unilateral symptoms); n = 30 H&YII (mild disease, no balance issues); n = 30 H&YIII (moderate disease, balance issues). Within the H&YII and III groups, we recruited a sub-group of n = 15 individuals who self-reported FOG within each group (n = 30 total with FOG). Self-reported FOG was based upon a question in the new Freezing of Gait Questionnaire.^30^ Participants were categorized as “freezers” if they had experienced such a feeling or episode over the past month. In addition, the presence of FOG was assessed in the laboratory during clinical examination, and if patients were seen with FOG but reported 0 on the FOG questionnaire, they were considered freezers.

Blinding was not possible for participants or assessors due to the use of specific equipment and/or instructions for the application of different cue strategies while walking. To minimize the risk of bias, recruitment and consent form did not mention potential superior effectiveness of a given cue strategy and instructions for application of cues were standardized.

### Clinical Assessment

Participants underwent a clinical assessment, which included collection of socio-demographic information and medical history, clinical, and cognitive tests. Motor signs related to PD severity were assessed with the motor section of the Movement Disorder Society Unified Parkinson’s Disease Rating Scale (MDS-UPDRS-III^31^). Global cognition was assessed with the Montreal Cognitive Assessment.^29^ Attention was assessed with a computerized button pressing test, involving simple and choice reaction time, and digit vigilance. Executive function was assessed using the Royall’s clock drawing^32^ and Trail-making Test Part B-A. Working memory and visuo-spatial ability were measured through seated forward digit span and judgement of line orientation tasks,^33^ respectively. Basic visual functions of visual acuity and contrast sensitivity were assessed using standardized charts (logMar and logCS). Fear of falling was measured using the Falls Efficacy Scale—International version.^34^

### Gait Assessment and Interventions

Participants walked back and forth over ~10 m for 2 minutes under several conditions while instrumented with fNIRS/EEG and inertial sensors. A baseline walking condition (i.e., walking at self-selected comfortable pace without cues) was performed first, followed by randomized cued walking conditions. Internal cue: participants were instructed to think about taking bigger steps while walking. External visual cues: transverse tapes (3 cm wide) were placed on the travel path and the distance between tapes was set at the individual step length (as measured during baseline walking); participants were instructed to step over the tapes. External auditory cues: participants were instructed to step in time to the beats of an electronic metronome set at their baseline cadence. External tactile cues: participants were instructed to step in time to metronome-like vibrations (also set at their baseline cadence) provided on their wrists through a pair of bracelets (Pulse, Soundbrenner). Specifically, we used baseline gait characteristics (e.g., cadence for auditory and tactile cueing; step length for visual cueing) to set the cue frequencies (distance or tempo), which aimed to focus on helping patients to reduce gait variability and/or enhance rhythm (sensory driven by external cues). For all walking conditions, participants stood still for 20 seconds before and after the walk bout.

### fNIRS/EEG: Cortical Brain Activity

A non-invasive and mobile combined fNIRS (OctaMon + Brite 24, Artinis Medical Systems, The Netherlands) and EEG system (SAGA 32-channel, TMSi, The Netherlands) recorded cortical brain activity while walking. The head cap included 32 electrodes and 28 optodes (consisting of 18 transmitters and 10 detectors). The fNIRS montage included 20 regular channels (inter-optode distance of 3 cm) and 6 short-separation channels (inter-optode distance of 1 cm). The fNIRS and EEG signals were synchronized and recorded at 50 Hz (Oxysoft) and 1000 Hz (SAGA Data Recorder 32+), respectively. A 3D-digitizer (Polhemus Patriot) was used to obtain coordinates of anatomical references and positions of electrodes and optodes. For EEG, electrode position from the digitizer (.xyz) was entered into EEGLAB channel location. For fNIRS, 3D-digitizer data were entered into Matlab via the software package NIRS-Statistical Parametric Mapping (NIRS-SPM, http://www.nitrc.org/projects/nirs_spm). The Spatial Registration routine (stand-alone NIRS, using 3D-digitizer) was used to find correspondence between scalp location where fNIRS measurements were performed and their underlying cortical surface where source signals were located. NIRS-SPM allowed the registration of fNIRS channel data onto the Montreal Neurological Institute standard brain space. EEG electrodes covered comprehensive cortical regions, whereas fNIRS optodes covered the prefrontal cortex, supplementary motor area, primary motor, somatosensory, and occipital cortices.

fNIRS data analysis was performed in line with our previous reliable fNIRS walking studies in PD.^5,25,35^ (1) signals were visually inspected; (2) artifacts were removed/attenuated by wavelet filtering; (3) oxygenated (HbO_2_) and deoxygenated (HHb) hemoglobin signals were low-pass filtered (cut-off 0.14 Hz); (4) short-separation channels were used to remove superficial blood flow from regular channels; (5) data were then baseline corrected by subtracting the mean of 20 seconds of initial standing period from the signal; (6) channels were averaged per regions of interest.^36,37^

EEG data analysis was performed in line with our previous data analysis.^26^ Initial signal processing was conducted using the EEGLAB toolbox (UC San Diego, Swartz Center for Computational Neuroscience (SCCN), USA) within MATLAB (Mathworks, USA).^38^ EEG data from separate 2-minute walking, and walking with each cueing condition, were concatenated within MATLAB, providing a 10-minute trial to process, which allowed multiple datasets across walking conditions in the same session to be used within independent component analysis (providing weights and source-locations). In brief, EEG data analysis consisted of the continuous EEG data being down-sampled from 2000 Hz to 500 Hz and a band-pass filter (1-200 Hz) was applied using the FIR filter function. The data were further filtered using the cleanLineNoise and clean_rawdata functions (for specific filtering specifications see Stuart et al^26^). Independent Component Analysis was performed on individual subject concatenated datasets (combined walking, visual, auditory, tactile, and internal cue).^39^ EEG signals were separated into maximally independent components (ICs) and the dipfit function derived single equivalent dipole that best represented the scalp topography of each IC using a boundary element electrical head model based on the Montreal Neurological Institute (MNI) template. ICs with residual scalp map variance above 15%, or whose dipole was located outside of the MNI head space, or had visual inspection of scalp topography or power spectrum that indicated artefactual source were excluded at the participant level. Additionally, ICs related to brain activity were identified using the ICLabel function (i.e., >70% likelihood of being brain activity rather than artefact).^40^ Joint measure IC pair distance measure was built using relative weights: power spectral density (1-200 Hz), 6; scalp map difference, 2; and dipole location difference, 12. The IC joint measure vectors were reduced to 10 dimensions by principle component analysis.

Clustering of EEG ICs was performed in line with our previous work,^26^ which used a K-means method to examine the Euclidean distance between ICs to find the cluster centroids, and to cluster the ICs nearest the centroids. Outlier components more than 3 standard deviations from any IC cluster centroid were removed from further analysis. Overall, 83 ICs were classified as outliers, which is a similar percentage to other previous EEG gait studies. The final number of clusters was determined through both dividing the number of ICs (n = 255) by the total number of subjects (n = 74) (i.e., 255/74 = 3.5), and by systematically starting with a small number of clusters (smallest in which activation properties were not merged across clusters) and increasing by 1 cluster at a time (i.e., if dipoles were separated into 2 but other IC properties or group differences remained consistent, then the maximum number of clusters was deemed exceeded). Clusters were retained for further analysis if they contained approximately half of the participants from each group, in line with previous work.

### Wearable Inertial Sensors: Gait

Wearable inertial sensors (Opal, APDM, USA) quantified spatiotemporal gait parameters at a sampling rate of 128 Hz. They were located at the sternum and pelvis levels, and bilaterally on the wrists, shanks, and feet of participants. Inertial sensors consisted of triaxial accelerometers, gyroscopes, and magnetometers, and were securely fixed to the participant’s body with Velcro straps. The inertial sensors and fNIRS/EEG system were synchronized through the Artinis PortaSync. Gait characteristics were extracted from the inertial sensors using the Mobility Lab software (Mobility Lab v2, APDM, USA).^41^

### Outcome Measures

The primary outcome measures for this study were cue-related changes in cortical activity during gait measured via fNIRS/EEG. Specifically, relative change in cortical HbO_2_ and PSD (log-transformed) of EEG clusters in 5 bandwidths (i.e., delta [1-4 Hz], theta [4-7 Hz], alpha [8-12 Hz], beta [14-30 Hz], and gamma [30-50 Hz] frequency bandwidths).

The secondary outcome measures were cue-related changes in gait characteristics, such as stride length, gait speed, arm range of motion, double support phase, and gait variability (standard deviation of consecutive strides); these are widely used to describe gait in people with PD.

### Statistical Analysis

Demographic, fNIRS, and gait data were analyzed using SPSS version 26 (The International Business Machines Corporation, USA), whereas EEG data statistical analysis was performed within the MATLAB EEGLAB toolbox. All statistical tests were carried out at the 5% 2-sided level of significance. Decision on data distribution followed the criteria proposed by Kim.^42^ Chi-Square test was used to compare gender across groups. One-way ANOVAs and Kruskal Wallis tests were used to compare demographic and clinical variables across groups, according to data distribution.

***fNIRS and gait data*:** Linear-mixed effects models were used to compare the primary and secondary outcomes across groups (i.e., H&I, H&YII, and H&YIII) and walking conditions (i.e., no cues and auditory, internal, tactile, and visual cueing). Primary and secondary outcomes with non-normal distribution were transformed before entering the linear-mixed effects models. Post hoc tests (adjusted for multiple comparisons) were used to localize differences when linear-mixed effects models revealed significant interactions or main effect of walking condition. The standardized response mean (SRM) was calculated to compare changes from walking without cues to walking with internal and external cues; SRM was interpreted as follows: trivial (<0.2), small (>0.2 and <0.5), moderate (>0.5 and <0.8), and large (>0.8).

***EEG data*:** EEG statistical analysis was carried out in line with our previous work.^26^ PSDs between groups (PD H&Y I, H&Y II, H&Y III) and conditions (Walk, Visual cue, Auditory cue, Tactile cue, Internal cue) with permutation statistics (2000 permutations) with a 95% confidence interval (*P* < .05). A False Discovery Rate (FDR) correction was applied to control for Type I error (multiple comparison). For overall effects, analysis involved permutation based repeated measures (3 × 5) ANOVA design (Group: H&YI, II, III, Condition: Walk, Visual, Auditory, Tactile, Internal). Within Group Effects: Post-hoc permutation based on-way repeated measures ANOVAs (1 × 5) were used to investigate differences between conditions (Walk, Visual, Auditory, Tactile, Internal) within each group (H&YI, II, III), with FDR adjustment. Between group effects: Post-hoc permutation based one-way repeated measures ANOVAs (1 × 3) were used to assess overall difference between the groups (H&YI, II, III) separately within each condition (Walk, Visual, Auditory, Tactile, Internal), with FDR adjustment. Additional separate paired permutation t-tests examined individual group differences (H&Y I vs II, H&Y I vs III, H&Y II vs III), with FDR adjustment.

## Results

### Participants

This study involved 80 people with PD. Table 1 shows that they had mild to moderate disease severity and preserved global cognition. ANOVA/Kruskal Wallis test revealed significant group differences for disease duration and MDS-UPDRS III score (i.e., H&I < H&YII < H&YIII), which were expected as participants were distributed into groups according to H&Y stage. Further, H&YII and H&YIII groups had greater levodopa equivalent daily dose than H&YI. Groups were matched for all other demographic variables.

**Table 1.** Participants Characteristics.

Variable	H&Y I	H&Y II	H&Y III	ANOVA/Kruskal Wallis *P*-value	Bonferroni Post-hoc
FoG status	−FoG = 20/+FoG = 0	−FoG = 15/+FoG = 15	−FoG = 15/+FoG = 15		
Male/female	M = 15/F = 5	M = 20/F = 10	M = 22/F = 8	.775	—
Age (years)	67.5 ± 7.5	68.7 ± 6.7	72.2 ± 9.7	.141	—
Height (cm)	168.8 ± 8.8	166 ± 21.4	167.7 ± 9.1	.939	—
Body mass (kg)	78.7 ± 11.8	85.9 ± 22.9	79 ± 15.2	.784	—
Years of education	12.5 ± 2.6	14.5 ± 3.4	12.9 ± 2.9	.068	—
Disease duration (years)	2.2 ± 1.9	5.5 ± 4.5	7.5 ± 5.7	<.001*	H&YI < H&YII < H&YIII
MDS-UPDRS III (score)	17.4 ± 7.5	38.1 ± 11.2	49.4 ± 12.7	<.001*	H&YI < H&YII < H&YIII
LEDD (mg/day)	314 ± 270.3	662.4 ± 418.6	706.1 ± 382.9	.001*	H&YI < H&YII−III
NFOG-Q (score)	0 ± 0	7.2 ± 8.7	7.5 ± 8.7	<.001*	H&YI < H&YII−III
MoCA (score)	27.4 ± 2.8	27.5 ± 1.9	25.9 ± 2.6	.030*	ns
Trail making test A (s)	42.8 ± 38.3	38.7 ± 13.8	47 ± 25.6	.144	—
Trail making test B (s)	102 ± 81.9	90.9 ± 59.7	127 ± 87.8	.093	—
Trail making test B-A (s)	59.2 ± 54.2	52.1 ± 50.4	80 ± 66.8	.091	—
CLOX 1 (score)	12.8 ± 1.2	12.8 ± 1.3	12.1 ± 1.7	.194	—
CLOX 2 (score)	13.5 ± 1	13.3 ± 1.3	12.9 ± 1.4	.199	—

* *P*<.05; Abbreviations: FoG, freezing of gait; H&Y, Hoehn & Yahr Rating Scale; LEDD, Levodopa equivalent daily dose; MDS-UPDRS, movement disorders society—Unified Parkinson’s Disease Rating Scale; MoCA, Montreal Cognitive Assessment; NFOG-Q, New Freezing of Gait Questionnaire; ns, non-significant.

No adverse events were reported. Two participants (one H&YII and one H&YIII) were unable to complete the walking protocol due to tiredness/fatigue; the non-completed walking conditions were treated as missing data points in the statistical analysis.

### fNIRS

*Group Effects:* Linear mixed effects models (walking condition X H&Y stage) revealed no significant interactions (*P* > .05) for relative HbO_2_ across multiple brain cortical regions of interest, indicating that H&Y stage did not influence cortical hemodynamic response to cueing in people with PD in H&YI to H&YIII (Figure S1; Table 2).

**Table 2.** Results for 2-Way Interactions (Group × Walking Condition) and Condition Main Effect for Relative HbO_2_ and Gait Outcomes, With Indications of Significant Post Hoc Paired Comparisons Relative to Walking Without Cues.

Outcomes	2-way interaction	Walking condition main effect	
	*F, P*	*F, P*	Post hoc tests
Relative HbO_2_			
Prefrontal cortex	0.544, .821	2.587, .041	NOcue < IC
Supplementary motor area	0.104, .999	0.898, .468	
Primary motor cortex	1.087, .377	2.677, .035	NOcue < VC, TC
Somatosensory cortex	0.990, .446	2.341, .057	
Visual cortex	0.710, .682	3.521, .010	NOcue < VC, AC, TC
Gait			
Speed	0.446, .893	30.821, <.001	NOcue < TC, IC, AC
Stride length	0.753, .644	74.174, <.001	NOcue < TC, IC, AC
Stride length variability	1.608, .126	17.794, <.001	NOcue > VC
Stride time variability	0.542, .823	5.000, <.001	NOcue < VC
Arm range of motion	0.312, .961	16.968, <.001	NOcue < IC, AC
Foot elevation at midswing	0.452, .888	44.765, <.001	NOcue < AC, VC
Double support phase	1.925, .057	5.360, <.001	NOcue > IC

Abbreviations: AC, auditory cueing; IC, internal cueing; NOcue, walking without cues; TC, tactile cueing; VC, visual cueing.

**Figure 1.**
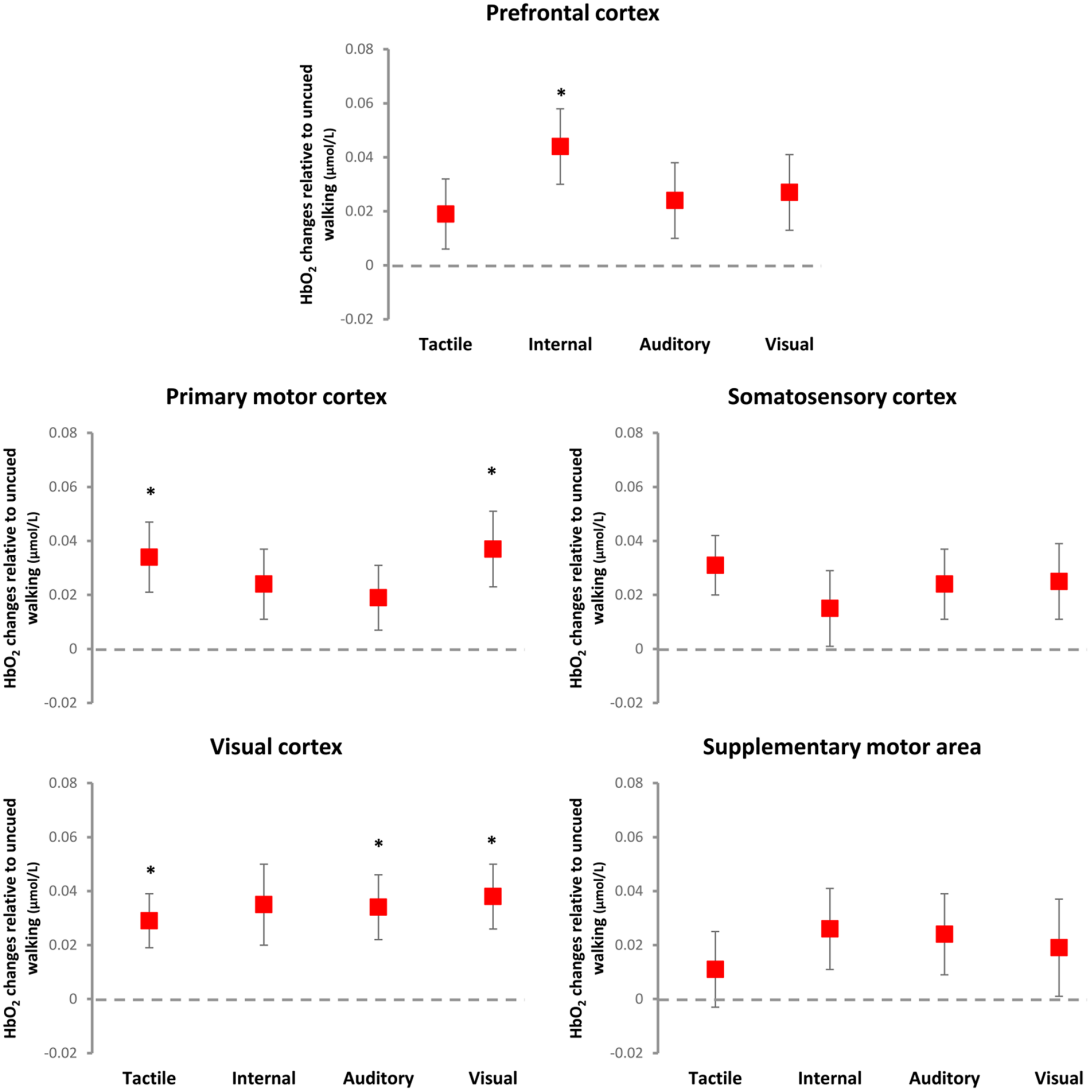
Illustration of cueing main effects on relative HbO_2_ levels (mean and standard error) relative to uncued walking (dashed line at zero). Internal cueing increased HbO_2_ levels in the prefrontal cortex; auditory cueing increased HbO_2_ levels in the visual cortex and; visual and tactile cueing increased HbO2 levels in the visual and primary motor cortices. * indicates significant difference relative to walking without cues.

*Cue Effects*: Linear mixed effects models revealed significant response to cueing in the PFC (F = 2.587, *P* = 0.041), primary motor (F = 2.677, *P* = 0.035), and visual cortices (F = 3.521, p = 0.010). Post-hoc tests adjusted for multiple comparisons showed the following significant differences relative to walking without cues (Figure 1; Table 2): internal cueing increased HbO_2_ levels in the PFC (SRM = 0.36); auditory cueing increased HbO_2_ levels in the visual cortex (SRM = 0.35), tactile cueing increased HbO_2_ levels in the visual (SRM = 0.30) and primary motor cortex (SRM = 0.31); and visual cueing increased HbO_2_ levels in the visual (SRM = 0.39) and primary motor cortices (SRM = 0.32).

### EEG

Overall, 255 ICs were retained across the 3 groups (H&YI, II, III), which were clustered into 3 active clusters (plus an outlier cluster of 83 ICs) that included 172 ICs, excluding the outlier cluster (83 ICs). Table S1 shows the cluster locations, number of subjects per cluster, corresponding Brodmann area (BA), and MNI co-ordinates for each cluster centroid. Clusters were located within the left somatosensory cortex, right parietal and left frontal cortices, indicating that these cortical areas were active during walking with, and without, all of the cueing modalities across the PD groups.

**Figure 2.**
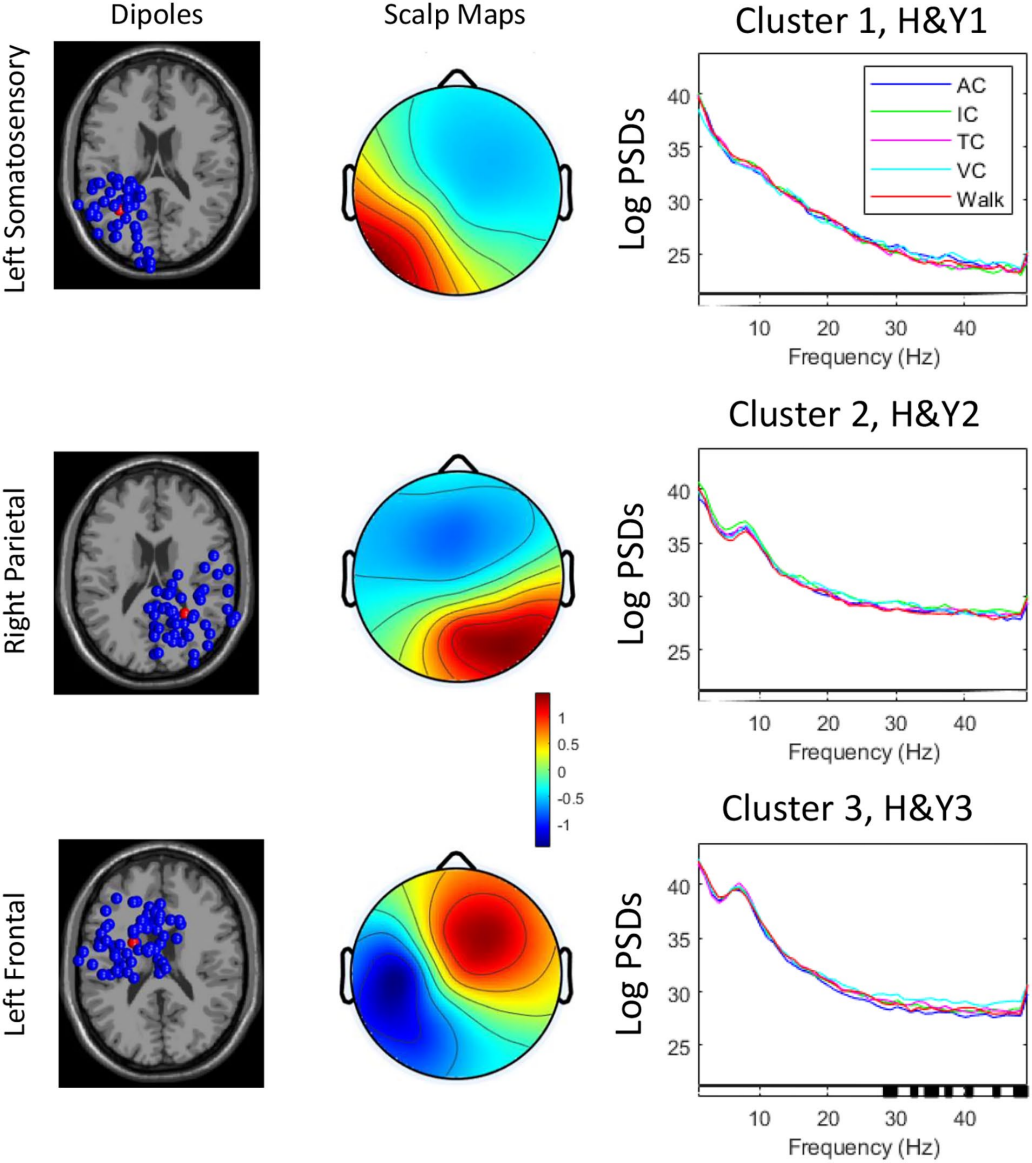
Power spectral densities in brain cluster locations in people with Parkinson’s disease across Hoehn & Yahr stages (H&YI, H&YII, H&YIII) when walking without or with cueing. AC: auditory cueing; IC: internal cueing; TC: tactile cueing; VC: visual cueing. [Dipole location, scalp maps, and LogPSDs, Levels of significance for within-group effects are shown at the bottom bar of each graph (significant findings are highlighted in black)].

*Within Group Effects:* Figure 2 shows the scalp maps and dipoles of the 3 clusters during walking with, and without, the cueing conditions within the 3 groups. No significant differences in PSD were observed for cued conditions relative to walking without cues (Figure 2). In H&YIII, PSD in gamma band in the left frontal cortex was higher in the visual cueing condition compared to auditory cueing condition (Figure 2), suggesting greater executive-attentional processing in the visual cueing condition.

**Figure 3.**
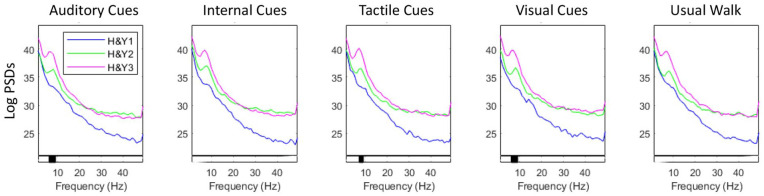
Power spectral density across different frequency bands in the parietal cortex for each group (Hoehn & Yahr: H&YI, H&YII, H&YIII) and walking condition. Levels of significance for between-group effects are shown at the bottom bar of each graph (significant findings are highlighted in black).

*Between group effects:* In the right parietal cortex, people with PD in H&YIII had higher alpha PSD than those in H&YI in the auditory, tactile, and visual cueing conditions (Figure 3).

### Gait Parameters

**Figure 4.**
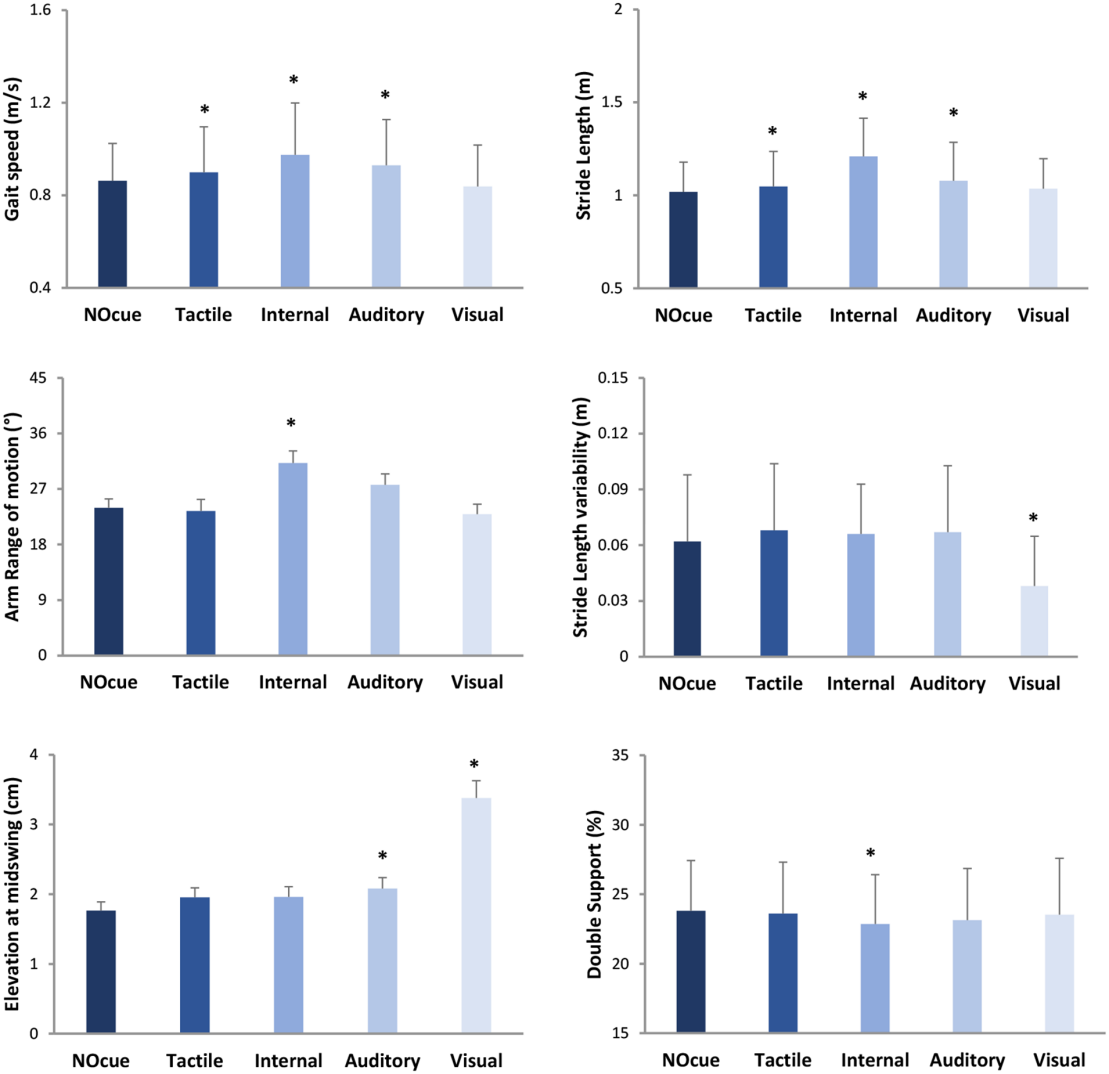
Illustration of cueing main effects on gait parameters (mean and standard deviation/error). Specific gait improvements were achieved with internal and visual cueing strategies, supporting a personalized, deficit-driven approach for cueing application. * indicates significant difference relative to walking without cues (NOcue).

Linear mixed effects models revealed no significant interactions between walking condition and disease stage for spatiotemporal gait parameters (*P* > .05), indicating that H&Y stage did not influence gait response to cueing in people with PD in H&YI to H&YIII (Table 2, Figure S2) (there was also no impact of FoG on cue response; Supplementary Table 1). Significant condition main effects (*P* < .05) were observed for multiple gait parameters (Table 2). Post-hoc tests adjusted for multiple comparisons showed the following significant differences relative to walking without cues (Figure 4): internal cueing increased gait speed (SRM = 0.83), stride length (SRM = 1.41), and arm range of motion (SRM = 0.56) and decreased the double support phase (SRM = 0.40); visual cues reduced stride length variability (SRM = 0.54) and increased foot elevation at midswing (SRM = 1.08) and stride time variability (SRM = 0.36); auditory cues increased gait speed (SRM = 0.47), stride length (SRM = 0.38), and foot elevation at midswing (SRM = 0.42); tactile cues increased gait speed (SRM = 0.40) and stride length (SRM = 0.30).

## Discussion

Gait response to internal and external cueing was not influenced by H&Y stage and, therefore, our hypothesis that H&YI would be more responsive to internal cueing was not confirmed. Instead, findings suggest that specific gait improvements (small to large effect sizes) are achieved with selective internal and external cueing strategies, supporting a personalized, deficit-driven approach for cueing application. Additionally, multiple brain cortical regions seem to be involved in the underlying mechanisms of gait improvement achieved with internal and external cues.

### Brain Response

fNIRS outcomes suggest that immediate brain cortical response to cueing was not influenced by mild to moderate PD stages. EEG analysis suggests a slightly different story as people with PD in H&YIII had higher alpha PSD in the parietal cortex than those in H&YI in the auditory, tactile, and visual cueing conditions (although no significant differences in PSD were observed for cued conditions relative to walking without cues). These EEG findings may indicate a slowing of EEG signal (i.e., greater power at lower frequency bands due to the reduction in excitatory signaling from the thalamus to sensorimotor cortical areas) or additional cognitive processing/demand in more advanced PD.^43^ Further, those in H&YIII seem to require greater executive-attentional processing in the visual cueing condition (relative to auditory cueing condition), given that PSD in gamma band in the left frontal cortex was higher in the visual cueing condition compared to auditory cueing condition.

Regarding cue-related changes in brain cortical activity (i.e., condition main effects), fNIRS outcomes identified small modality-specific responses (effect sizes ranged from 0.30 to 0.39). Relative to walking without cues, only internal cueing increased activity in the PFC, suggesting increased executive-attentional demand for internal cueing; in other words, an executive-attentional strategy can compensate for reduced automaticity in the control of walking in PD, which is well documented in the literature.^4,5,44,45^ External cueing modalities increased activity in areas associated with sensorimotor processing (i.e., primary motor and visual cortices), indicating enhanced sensorimotor processing and/or sensory feedback as mechanisms underpinning gait improvements achieved with external cueing.

### Gait Response

Immediate gait response to cueing was not influenced by PD stage (HY&I-III). Although several gait characteristics responded to both internal and external cueing, the response was not different across H&YI to H&YIII. These findings partially disagree with physical therapy guidelines suggesting that different cueing strategies may be useful for gait improvement at specific PD stages.^14^ For example, while the traditional recommendation is that in the later stages of PD (H&Y II-IV) only external cues are effective,^15,16^ our findings suggest that people with PD in H&Y II-III can also benefit from internal cueing.^17^

Given that specific gait improvements were achieved with different cueing strategies, findings support a deficit-driven approach for choosing the most appropriate cueing strategy for each individual. Clinical practitioners should consider the observed effect sizes to inform their decision on which cueing strategy to apply. Large effect sizes (SRM > 0.8) were only observed with the application of internal cueing (for gait speed and stride length) and external visual cueing (for foot elevation at midswing). Moderate effect sizes (SRM = 0.5-0.8) were also only achieved with internal cueing (for arm range of motion) and external visual cueing (for stride length variability). Therefore, internal and visual cueing seem more effective than other cueing strategies, which had only small immediate effects on gait parameters.

### Strengths and Limitations

A key strength of the current study is the concurrent assessment of movement and cortical activity in response to several cueing strategies. This approach allowed us to better understand both behavioral responses and underlying cortical mechanisms. Further, by directly comparing the responses to different cueing modalities, we were able to identify the ones that provide larger benefits to patients. Studying brain activity response with both fNIRS and EEG allowed insights into cue response that would not have been possible with only one technology. Due to the differences in sensor placement and physiological processes being measured by the technologies (e.g., spatial resolution fNIRS = 2-3 cm, EEG 5-9 cm, temporal resolution fNIRS = 1-50 Hz, EEG = 1-2000 Hz), we expected that findings would be complementary and allow more comprehensive/accurate understanding of cue response, like other EEG-fNIRS studies.^46,47^

Limitations include the lack of blinding. Clinical application of cueing strategies involves equipment or specific instructions to patients, and therefore we were unable to blind participants or assessors. To minimize the impact of lack of blinding on findings we applied a random order to cueing strategies, and participants were not given information about potential superior effectiveness of different cue strategies, and standardized instructions were given. While H&Y scale is the most widely used method to differentiate PD stages, future studies may consider other classification methods that may be more granular. Finally, because this study is focused on the immediate response to cues (i.e., single exposure), interpretations cannot be generalized to long-term application and potential adaptation to cues.

## Conclusions

Findings suggest that people with PD across H&Y stages I to III similarly improve walking in response to internal and external cues. Specific gait improvements (small to large effect sizes) are achieved with different cueing strategies. Regarding the underlying mechanisms, cue-related changes in brain cortical activity indicate that walking with specific cues may be underpinned by greater cognitive, motor and sensory processing within selective brain regions, which may be influenced by PD stage (H&YI-III).

## Supplemental Material

sj-docx-1-nnr-10.1177_15459683251351876 – Supplemental material for Effects of Internal and External Cues on Brain Activity and Gait in Parkinson’s Disease: Findings From BARC-PD Supplemental material, sj-docx-1-nnr-10.1177_15459683251351876 for Effects of Internal and External Cues on Brain Activity and Gait in Parkinson’s Disease: Findings From BARC-PD by Rodrigo Vitorio, Rosie Morris, Lisa Graham, Julia Das, Richard Walker, Claire McDonald, Martina Mancini and Samuel Stuart in Neurorehabilitation and Neural Repair

sj-docx-2-nnr-10.1177_15459683251351876 – Supplemental material for Effects of Internal and External Cues on Brain Activity and Gait in Parkinson’s Disease: Findings From BARC-PD Supplemental material, sj-docx-2-nnr-10.1177_15459683251351876 for Effects of Internal and External Cues on Brain Activity and Gait in Parkinson’s Disease: Findings From BARC-PD by Rodrigo Vitorio, Rosie Morris, Lisa Graham, Julia Das, Richard Walker, Claire McDonald, Martina Mancini and Samuel Stuart in Neurorehabilitation and Neural Repair

sj-docx-3-nnr-10.1177_15459683251351876 – Supplemental material for Effects of Internal and External Cues on Brain Activity and Gait in Parkinson’s Disease: Findings From BARC-PD Supplemental material, sj-docx-3-nnr-10.1177_15459683251351876 for Effects of Internal and External Cues on Brain Activity and Gait in Parkinson’s Disease: Findings From BARC-PD by Rodrigo Vitorio, Rosie Morris, Lisa Graham, Julia Das, Richard Walker, Claire McDonald, Martina Mancini and Samuel Stuart in Neurorehabilitation and Neural Repair

sj-docx-4-nnr-10.1177_15459683251351876 – Supplemental material for Effects of Internal and External Cues on Brain Activity and Gait in Parkinson’s Disease: Findings From BARC-PD Supplemental material, sj-docx-4-nnr-10.1177_15459683251351876 for Effects of Internal and External Cues on Brain Activity and Gait in Parkinson’s Disease: Findings From BARC-PD by Rodrigo Vitorio, Rosie Morris, Lisa Graham, Julia Das, Richard Walker, Claire McDonald, Martina Mancini and Samuel Stuart in Neurorehabilitation and Neural Repair
