# Supplementary material for: Effects of Internal and External Cues on Brain Activity and Gait in Parkinson’s Disease: Findings From BARC-PD
Source: Neurorehabil Neural Repair. 2025 Jul 13;39(10):826–38. doi: 10.1177/15459683251351876 (PMC12476477; doi:10.1177/15459683251351876)
Supplement: sj-docx-1-nnr-10.1177_15459683251351876 – Supplemental material for Effects of Internal and External Cues on Brain Activity and Gait in Parkinson’s Disease: Findings From BARC-PD [file sj-docx-1-nnr-10.1177_15459683251351876.docx]

**Figure S1**. Illustration of relative HbO_2_ levels (mean and standard error) for each HY group and walking condition. Linear mixed-effects models revealed non-significant interactions (p>0.05) between group and walking condition for all brain cortical regions of interest.
