# Supplementary material for: Effects of Internal and External Cues on Brain Activity and Gait in Parkinson’s Disease: Findings From BARC-PD
Source: Neurorehabil Neural Repair. 2025 Jul 13;39(10):826–38. doi: 10.1177/15459683251351876 (PMC12476477; doi:10.1177/15459683251351876)
Supplement: sj-docx-2-nnr-10.1177_15459683251351876 – Supplemental material for Effects of Internal and External Cues on Brain Activity and Gait in Parkinson’s Disease: Findings From BARC-PD [file sj-docx-2-nnr-10.1177_15459683251351876.docx]

**Figure S2**. Selected gait parameters (mean and standard deviation). Linear mixed effects models revealed no significant interactions between walking condition and H&Y stage (p>0.05), indicating that H&Y stage do not influence gait response to cueing in people with PD in H&YI to H&YIII.
