# Supplementary material for: Effects of Internal and External Cues on Brain Activity and Gait in Parkinson’s Disease: Findings From BARC-PD
Source: Neurorehabil Neural Repair. 2025 Jul 13;39(10):826–38. doi: 10.1177/15459683251351876 (PMC12476477; doi:10.1177/15459683251351876)
Supplement: sj-docx-3-nnr-10.1177_15459683251351876 – Supplemental material for Effects of Internal and External Cues on Brain Activity and Gait in Parkinson’s Disease: Findings From BARC-PD [file sj-docx-3-nnr-10.1177_15459683251351876.docx]

**Supplementary Table 1**. Results for 2-Way Interactions (FoG status * Walking condition) and condition main effect for gait outcomes, with indications of significant post hoc paired comparisons relative to walking without cues.

| **Gait outcomes** | **2-way interaction** |  | **Walking condition main effect** | |
| --- | --- | --- | --- | --- |
|  | ***F, p*** |  | ***F, p*** | ***Post hoc tests*** |
| Speed | .166, .955 |  | 30.466, <.001 | NOcue < TC, IC, AC |
| Stride length | .271, .896 |  | 73.096, <.001 | NOcue < TC, IC, AC |
| Stride length variability | .343, .848 |  | 18.463, <.001 | NOcue > VC |
| Stride time variability | 1.458, .218 |  | 3.908, .005 | NOcue < VC |
| Arm range of motion | .272, .896 |  | 23.158, <.001 | NOcue < IC |
| Foot elevation at midswing | .952, .435 |  | 39.814, <.001 | NOcue < AC, VC |
| Double support phase | .196, .940 |  | 5.087, <.001 | NOcue > IC |

AC: auditory cueing; IC: internal cueing; NOcue: walking without cues; TC: tactile cueing; VC: visual cueing.
