# Supplementary material for: Effects of Internal and External Cues on Brain Activity and Gait in Parkinson’s Disease: Findings From BARC-PD
Source: Neurorehabil Neural Repair. 2025 Jul 13;39(10):826–38. doi: 10.1177/15459683251351876 (PMC12476477; doi:10.1177/15459683251351876)
Supplement: sj-docx-4-nnr-10.1177_15459683251351876 – Supplemental material for Effects of Internal and External Cues on Brain Activity and Gait in Parkinson’s Disease: Findings From BARC-PD [file sj-docx-4-nnr-10.1177_15459683251351876.docx]

**Table S1.** Localizations of cluster centroids are shown in Talairach coordinates along with the corresponding nearest gray matter locations and Brodmann areas

|  |  |  | **Centroid Co-ordinates** | | |  |  |
| --- | --- | --- | --- | --- | --- | --- | --- |
|  | **No. of Subjects** | **No. of ICs** | **X** | **Y** | **Z** | **BA** | **Grey Matter Location** |
| ***Cluster 1***  Left Somatosensory Cortex | Total = 40  H&Y I = 12  H&Y II = 13  H&Y III = 15 | 50 | -33 | -55 | 13 | 1, 2, 3 | Postcentral Gyrus |
| ***Cluster 2***  Right Parietal Cortex | Total = 42  H&Y I = 10  H&Y II = 15  H&Y III = 17 | 59 | 26 | -50 | 45 | 7 | Precuneus |
| ***Cluster 3***  Left Frontal Cortex | Total = 44  H&Y I = 12  H&Y II = 18  H&Y III = 14 | 63 | -23 | 2 | 50 | 24, 46 | Cingulate Gyrus  Medial Frontal Gyrus |
|  |  |  |  |  |  |  |  |

*[IC; independent components, BA; Broadman Areas]*
